# *Aglaomorpha quercifolia* (L.) Hovenkamp & S. Linds a Wild Fern Used in Timorese Cuisine †

**DOI:** 10.3390/foods10010087

**Published:** 2021-01-04

**Authors:** Hermenegildo R. Costa, Inês Simão, Helena Silva, Paulo Silveira, Artur M. S. Silva, Diana C. G. A. Pinto

**Affiliations:** 1 LAQV-REQUIMTE & Department of Chemistry, Campus de Santiago, University of Aveiro, 3810-193 Aveiro, Portugal; hrcosta@ua.pt (H.R.C.); artur.silva@ua.pt (A.M.S.S.); 2 CESAM—Centre for Environmental and Marine Studies & Department of Biology, Campus de Santiago, University of Aveiro, 3810-193 Aveiro, Portugal; ines.simao@ua.pt (I.S.); hsilva@ua.pt (H.S.); psilveira@ua.pt (P.S.); 3 Faculty of Education, Arts and Humanities, National University Timor Lorosa’e (UNTL), Avenida Cidade de Lisboa, Dili, East Timor

**Keywords:** *Aglaomorpha quercifolia*, GC-MS profile, rhizome, leaves, *n*-hexane extract, fatty acids, terpenoids, linolenic acid, hop-16-ene

## Abstract

*Aglaomorpha quercifolia* (L.) Hovenkamp & S. Linds is an extensively used species in traditional medicinal systems in several areas of the world due to some important medicinal properties such as antioxidant, antibacterial, analgesic, and anti-inflammatory activities. In East Timor, different parts of this fern are used either as remedies or as food. The ingestion of a broth made from its rhizome improves lactation, and young fronds of this fern are boiled and eaten with rice by the locals. Nevertheless, its chemical profile is far from being established. The present work aims to establish the chemical profile of both rhizomes and leaves *n*-hexane extracts by Gas Chromatography- Mass Spectrometry (GC-MS). The results showed the leaves richness in fatty acids with interesting nutritional values (ω−6/ω−3 = 0.68, AI = 0.59, TI = 0.30), being linolenic acid (253.71 ± 0.93 mg/g dry leaves) and palmitic acid (237.27 ± 0.59 mg/g dry leaves) the significant compounds in the extract. Whereas the rhizome extract is mostly rich in terpenoids, such as steroid, cycloartane, and hopanoid derivatives, being hop-16-ene (166.45 ± 0.53 mg/g dry rhizome) and β-sitosterol (50.76 ± 0.11 mg/g dry rhizome) the major compounds. Several compounds are reported for the first time in the species, and the data herein reported contributes to confirming the species nutritional value.

## 1. Introduction

*Aglaomorpha quercifolia* (L.) Hovenkamp & S. Linds., [syn. (*Drynaria quercifolia* (L.) J.Sm., J. Bot. (Hooker)] [1] is an epiphytic, occasionally epilithic medicinal pteridophyte with a short-creeping rhizome, dimorphic fronds, and pinnatifid lamina. It belongs to the Polypodiaceae family, which includes 65 genus and 165 species worldwide [2]. *A. quercifolia* occurs in primary and secondary forests, savannas, and plantations (such as rubber and coconut), but it can also be found along sideroads [3].

*Aglaomorpha quercifolia* is an extensively used species in traditional medicinal systems in several areas of the world. For example, in India, it is used by tribal communities to cure several different conditions. The juice produced from the rhizome and fronds is taken for body pain [4] and intestinal worms [5]. This fern is also used to treat throat infections, tuberculosis [6], jaundice, dysentery, and typhoid fever [7]. In Bangladesh, several parts of this fern have been used to treat jaundice [8], gonorrhea [9], diabetes [10], and malaria [11]. In East Timor, this fern rhizome is used either as a remedy to treat stomach pain or as food. It is believed that the ingestion of a broth made from its rhizome helps young moms producing more milk [12]. The rhizome is also consumed as a tea, and the young fronds of this fern are boiled and eaten with rice by the locals [12]. A paste made from the rhizome is massaged onto people with malaria [13].

The above-mentioned vast range of traditional uses concerning *A. quercifolia* are most certainly related to its many medicinal properties, such as antioxidant [14], antibacterial [15], analgesic [16], anti-inflammatory [17], anthelmintic [18], antipyretic [19], and antirheumatic [20]. These medicinal properties must be directly related to the secondary metabolites produced by the plant. Although these metabolites are not essential to the plant’s life, they contribute directly to its fitness [21] and the interactions between the organism and the environment [22]. It is known that several classes of secondary metabolites with different functions in the plant are responsible for the plant’s traditional medicine applications.

In terms of its secondary metabolites, *A. quercifolia* is not an extensively studied species. Studies involving methanolic or ethanolic extracts of the whole plant or the rhizome revealed the identification of several compounds [23,24,25]. Due to the use of polar solvents in the extraction, several reported compounds are polyphenolic, and others seemed not to be secondary metabolites. So, concerning the use of *A. quercifolia* in traditional medicine and nutrition, further studies focused on the plant’s lipophilic constituents are needed to support its traditional use and possible medicinal properties.

Thus, this study aimed to establish the GC-MS profile of both *A. quercifolia* rhizomes and leaves *n*-hexane extract and simultaneously confirm the species’ nutritional and medicinal value.

## 2. Materials and Methods

### 2.1. Plant Collection

Specimens of *Aglaomorpha quercifolia* (L.) Hovenkamp & S. Linds. were collected from Dare (Vera Cruz, Dili, East Timor) in July 2016. A voucher specimen was identified by the plant taxonomist Paulo Silveira and deposited in the Herbarium of the Department of Biology, University of Aveiro, Portugal (AVE), under the reference number AVE7891 (Costa HR 87).

Several plants were collected (around 100 plants), the plant rhizome and leaves were separated, washed, and dried at room temperature for 7 days. Six samples having ten plant parts were powdered using an electrical blender.

### 2.2. Extracts Preparation

For the extraction in *n*-hexane, the amount of plant was determined on a precision scale, RADWAG WLC 6/A2, with a precision of 0.1 g. In the process, 100 mL of *n*-hexane for each 10 g of the plant was used. In Table 1, the weights of plant powder and the amount of *n*-hexane are listed. The plant parts were put into an Erlenmeyer flask, a magnetic stirrer was added, and the flasks were placed on a stirring plate. The *n*-hexane was added, see volume in Table 1, and the stirring was started at a speed of 600 rpm. Because some compounds can decompose under the light influence, the flasks were covered with aluminum foil in advance.

One extraction ran for 48 h, and the solvent was changed at least twice until no intensive color and increase of extraction weight of the solvent occurred. After an extraction cycle, the solvent was filtered and evaporated. After the three extraction cycles, the extracts were dried until mass consistency before further usage. The procedure was repeated for two more samples, and the average weights of *n*-hexane dried extracts (after constant mass) are shown in Table 1.

### 2.3. Standards and Reagents

Several pure compounds were used as standards to ensure the identification of the phytochemicals and to perform the calibration curves for quantification purposes. Tetradecane (99%), hexadecane (99.5%), tetracosane (99%), octadecane (99%), 1-monopalmitin (>99%), β-sitosterol (98%), lupeol, 5α-cholestan-3β-ol (99%), D-mannitol (98%), 1-tetradecanol (98%), sorbitol (99%), D-(+)-galactose (>99%), D-(+)-mannose (>99%), D-(+)-xylose (>99%), D-(−)-ribose (>99.5%), D-fructose (99%), sucrose (>99%), maltose (>98%), stigmasterol (97%), cycloartenol (>99%), campesterol (95%), lupeol (99%), ursolic (98%), oleanolic (98%), palmitic (≥99%) and stearic (99%) acids, were purchased from Sigma-Aldrich (St. Louis, MO, USA). Malonic acid (98%), linoleic acid (≥99%), and glycerol (>99%) were purchased from BDH analytical chemicals (London, UK), D-(−)-cellobiose (>98%), α- and β-tocopherol (98%) from Merk (Darmstadt, Germany), and D-(−)-arabinose (>99%) from Fluka (Bucharest, Romania) while, eicosane, docosane, hexatriacontane, and *n*-paraffin mixtures (C5–C8, C7–C10, C10–C16, C18–C24, C24–C36, C25–C35) were supplied by Supelco Inc. (Bellefonte, PA, USA).

For extraction, hexane pro-analysis (p.a) was used while dichloromethane (p.a.) (DCM) was employed to dissolve the extracts. Pyridine p.a., *N*, *O*-bis(trimethylsilyl)trifluoroacetamide (BSTFA) (99%) and trimethylsilyl chloride (TMSCl) (99%) (Sigma-Aldrich) were applied in the sample derivatization by silylation.

### 2.4. Gas Chromatography—Mass Spectrometry Analysis

The GC-MS analysis of the *n*-hexane extracts was done using a Shimadzu GCMS-QP2010 Ultra system equipped with autosampler AOC-20i, ion source: electronic impact high-performance Quadrupole mass filter. Separation of the compounds was carried out in a DB-5J&W capillary column (30.0 m in length × 0.25 mm in diameter × 0.25 µm thickness of the film). The spectroscopic detection from the mass spectrometer utilized 0.1 kV electron ionization. Helium was used as a carrier gas with a column flow of 1.18 mL/min. GC-injection temperature was set to ϑ = 320 °C and split ratio of 50 was applied to an injection volume of 1 µL. The mass spectrometer ion source temperature was set to ϑ = 250 °C and the interface temperature to ϑ = 300 °C.

The extracts were weight with approximately m = 20 mg on an analytical scale into a tube. DCM was used as a solvent and *n*-tetracosane as an internal standard, added to the tube with 1 mL in total. The extracts were then dissolved in an ultra-sonic bath. For the silylation, 250 µL pyridine, 250 µL BSTFA, and 50 µL TMSCl were added. The mixture was maintained in a water bath at ϑ = 70 °C for 45 min being the hydroxy and the carboxy groups present in each secondary metabolite converted to trimethylsilyl (TMS) ethers and esters, respectively. Afterwards were injected twice in the GC-MS apparatus. The silylation reagents quantity, the water bath temperature and the reaction time were previously optimized to ensure a total conversion of all compounds with hydroxy groups into the correspondent TMS derivatives.

The chromatographic conditions were as follows: start time of record at 6.5 min; initial temperature ϑ = 90 °C, hold for 4.00 min; temperature rate, 16 °C/min up to ϑ = 180 °C; temperature rate, 6 °C/min up to ϑ = 250 °C; followed by temperature rate, 3 °C/min up to ϑ = 300 °C and then hold for 5.00 min.

From the total ion chromatogram, the peaks were identified by comparing their mass spectra with the mass spectra libraries NIST 2014, NIST 2008, and WILEY 2007, and with mass spectra fragmentation published in the literature [26,27,28,29,30]. If possible, it was also compared with the retention time and mass spectra of standard compounds injected in the same chromatographic conditions. Furthermore, identification of some compounds was done using the retention index relative to *n*-alkanes (C5–C36) injected in the same chromatographic conditions and using the Equation (1). Where z is the number of carbon atoms in the alkane before the unknown compound and Z the number of the longer alkane. The retention time is t_r_ [31]:

(1) $$I=100\cdot \left[z+\left(Z-z\right)\cdot \frac{t_{r\left(\mathrm{unknown}\right)}-t_{r\left(z\right)}}{t_{r\left(Z\right)}-t_{r\left(z\right)}}\right].$$

For quantification purposes, four independent replicates of each sample were submitted to silylation procedure and each one injected in duplicate. The internal standard method was applied and the amount of metabolites present was achieved from the calibration curves obtained with the most closed pure standard compounds available or its TMS derivatives (if they have hydroxy groups). All the injected samples and standards solutions contain a fixed quantity of internal standard (tetracosane). The calibration curves were obtained by injection of at least six different concentrations (5 µg mL^−1^ to 1.5 mg mL^−1^) and the detection and quantification limits (LOD and LOQ, respectively) were determined from the parameters of the calibration curves represented in Table 2 (LOD = 3 standard deviation/slope and LOQ = 10 standard deviation/slope). Values of correlation coefficients confirmed linearity of the calibration plots (Table 2). The concentrations of the standards were chosen in order to guarantee the quantification of each compound in the samples by intrapolation in the calibration curve. The results were expressed in mg of compound/g of extract, as mean values ± standard deviation (MV ± SD) of four independent analyses.

### 2.5. Statistics

Independent replicates of each sample were analyzed and each aliquot was injected twice. The presented results are the average of four concordant values obtained for each sample (less than 5% variation between injections of the same aliquot and between aliquots of the same sample) and expressed as mean values ± standard deviation (MV ± SD). One-way analysis of variance (ANOVA) followed by Duncan’s multiple-range test were performed using the GraphPad Prism version 7 for Windows (Graphpad Software, Inc.) to compare the results of each independent replicates. A *p*-value lower than 0.0001 was considered statistically significant in all analyses.

## 3. Results and Discussion

**Figure 1:**
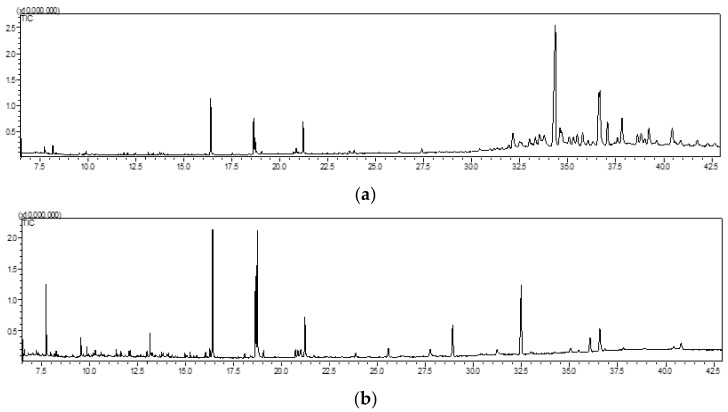
Total ion chromatogram (TIC) of *A. quercifolia* rhizomes (**a**) and leaves (**b**) *n*-hexane extracts with time in minutes.

Although the known use of *A. quercifolia* in traditional medicine [12,13] and a few studies involving GC-MS analysis were reported [23,24,25], this species still is, from the chemical profile point of view, underexplored. So, both rhizome and leaves were extracted with *n*-hexane at room temperature, aiming to obtain the lipophilic profile. Although this type of extraction was not reported for this species, our experience indicates that low extraction yields, such as the ones herein reported (Table 3), are typical in plants growing in warm environments [32]. Nevertheless, it was possible the identification and quantification, using GC-MS, of the major compounds present in both rhizome and leaves extracts, whose chromatograms demonstrate the richness in lipophilic compounds, although some only in traces (Figure 1). This analysis allowed identifying a total of 59 compounds, 31 in rhizome extract, and 34 in leaves extract. These were distributed through several chemical families, explicitly amid fatty acids, short-chain carboxylic acids, carbohydrates, terpenoids, alkanes, and alcohols. The retention time, identification, and content of each compound in mg/g of dried rhizome or leaves ± standard deviation of each species are presented in Table 3.

It is evident that the rhizomes and leaves lipophilic profiles are not very rich in secondary metabolites (Figure 1). Different results were observed regarding the mass of compounds identified in each extract, 70.3% in rhizomes and 99.6% in leaves. However, the percentage of compounds not identified by GC-MS was, therefore, approximately 30% for rhizome and less than 1% for leaves.

**Figure 2:**
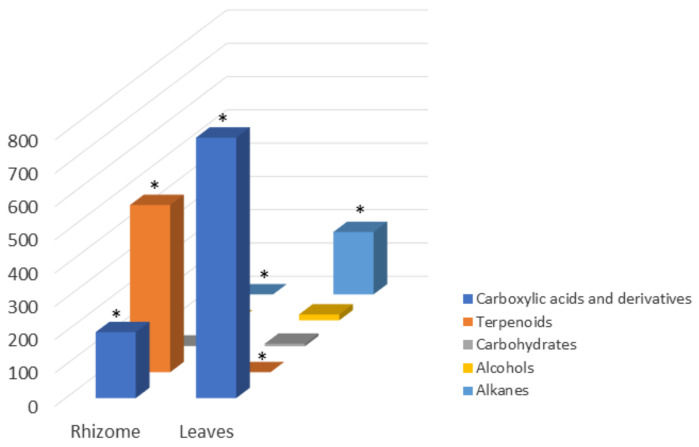
Graphical presentation of the total amount of each class of compounds for the two studied plant parts. * Statistically different (Tukey’s test) *p* < 0.001.

Regarding rhizomes extract, the most abundant chemical family present is terpenoids (50.2%), which included diterpenes, triterpenes, and steroids (Table 3, Figure 2). The rhizome chemical profile was dominated by hop-16-ene (166.45 ± 0.53 mg/g dry rhizome) and β-sitosterol (50.76 ± 0.11 mg/g dry rhizome). The second major chemical family identified in this plant part lipophilic extract was carboxylic acids and derivatives, summing a total of 19.8% of the identified compounds (Figure 2). The fatty acids palmitic and linoleic dominated the family with 107.65 ± 0.12 mg/g dry rhizome and 60.97 ± 0.08 mg/g dry rhizome, respectively (Table 3).

Leaves extract showed more diversity in the chemical families present and their representativity (Figure 2). The major chemical family in its lipophilic extract, representing 78.3% of the identified compounds, was the carboxylic acids and derivatives. In addition, among the identified compounds, palmitic, linolenic, and linoleic acids were the ones found in the highest amount, respectively 237.27 ± 0.59 mg/g dry leaves, 253.71 ± 0.93 mg/g dry leaves, and 153.81 ± 0.11 mg/g dry leaves (Table 3). Alkanes were the second major class observed, representing 18.7% of the identified compounds (Figure 2), being the *n*-tritetracontane found in significant quantities (131.3 ± 0.64 mg/g dry leaves). The remaining chemical families represent 0.2% and 2.5% of the rhizome and leaves’ identified compounds, respectively (Figure 2).

Alkanes represent more than 70% of the wax cuticle constitution, which is indispensable to prevent water loss [33], so their detection in *A. quercifolia* leaves seems quite normal. *n*-Alkanes are easily distinguishable by their mass spectrum due to the first fragment ion peak, represented by [M − 29]^+^ ion (loss of a ^•^CH_2_CH_3_), the base peak occurs at 43 or 57 *m*/*z*, and peaks differing by 14 *m/z* units (e.g., 43, 57, 71, 85, etc.) are present [34]. Nevertheless, their identification was possible mainly using pure standards and comparing with GC-MS databases.

A detailed analysis of Table 3 shows that *A. quercifolia* leaves are incredibly rich in fatty acids, both saturated fatty acids (SFA) and polyunsaturated fatty acids (PUFA), from which, respectively, palmitic acid (237.27 ± 0.59 mg/g dry leaves) and linolenic acid (253.71 ± 0.93 mg/g dry leaves) can be highlighted (Table 3). The plant rhizome also presents a high quantity of palmitic acid (107.65 ± 0.12 mg/g dry rhizome), contributing to a higher amount of SFA compared to PUFA (Table 3). However, it should be emphasized that recent evidence points out that SFA in the human diet may not have such an adverse health effect [35]. In the case of leaves, it is evident that some nutritional indexes, such as ω−6/ω−3 (total of omega-6 acids/total of omega-3 acids ratio), atherogenicity index (AI), and thrombogenicity index (TI), present values (ω−6/ω−3 = 0.68; AI = 0.59; TI = 0.30) that suggest nutritional and health-promoting values [36,37].

**Figure 3:**
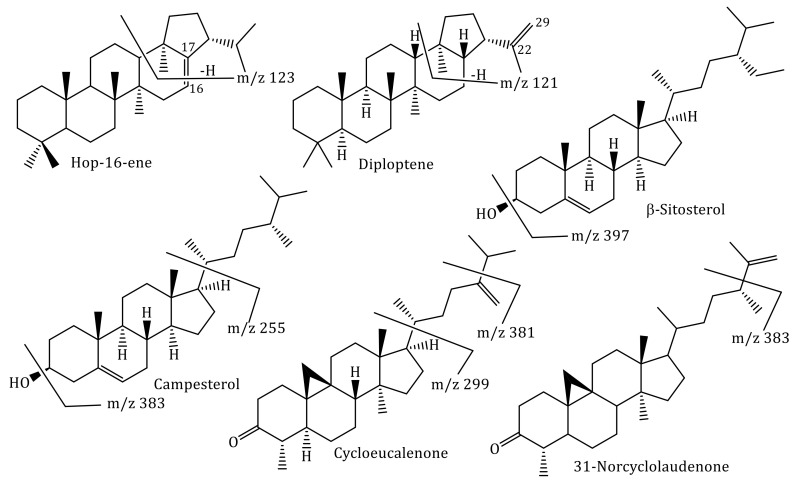
Chemical structure of some of the identified terpenoids and some of the most significant fragments.

Concerning the rhizome, it is evident its richness in terpenoid derivatives (Figure 2), from which cycloartane, hopanoid, and phytosterol derivatives can be highlighted and being the major compounds hop-16-ene (166.45 ± 0.53 mg/g dry rhizome), β-sitosterol (50.76 ± 0.11 mg/g dry rhizome), diploptene (48.01 ± 0.13 mg/g dry rhizome), cycloeucalenone (26.75 ± 0.02 mg/g dry rhizome), campesterol (25.64 ± 0.12 mg/g dry rhizome), and 31-norcyclolaudenone (21.05 ± 0.06 mg/g dry rhizome) (Table 3 and Figure 3).

Some of the terpenoids found in *A. quercifolia* rhizomes are commonly found in plants, including some phytosterols found in our previous works [32,38]. Cycloartane triterpenoids’ natural occurrence is also vast through the plant kingdom [39], being cycloartenol the most recognized due to its rule in the phytosterols biosynthesis [40] and consequently in the regulation of important plant functions [41]. The occurrence of this type of triterpenoids in *A. quercifolia* rhizomes (Table 3) may explain the effect that the rhizome intake promotes on young moms. Actually, there are references to the use of several plants to improve breastfeeding [42,43,44] and evidence that terpenoids are also involved [42,43].

Lastly, it seems imperative to mention the hopanoids triterpenes, which are the major class found in *A. quercifolia* rhizomes, mainly due to the presence of hop-16-ene (Table 3 and Figure 3). This type of triterpenes frequently occurs in ferns and was described for the first time by John Hope, a British botanist [45,46]. Several hopanoids, including hop-16-ene and diploptene (Figure 3), were found in *Davallia mariesii* rhizomes, and our results are identical to those previously reported [47]. The mass fragments confirm, in particular, the position of the double bond at C16 = C17 in hop-16-ene and C22 = C29 in diploptene (Figure 3). Moreover, the NMR data showed a proton sign at δ 5.28 ppm correlated with a carbon sign at δ 115.6 ppm characteristics of the hop-16-ene vinylic proton and carbon. Whereas in the case of diploptene, it is possible to detect the vinylic proton and carbon, respectively, at δ 4.78 and δ 109.9 ppm, data that are similar to the ones previously reported [47]. It is also important to highlight that the hop-16-ene DEPT 90 and DEPT 135 spectra confirm the presence of 6 methine, 10 methylene, and 8 methyl carbons, data that also ensure the proposed structure (Figure 3).

## 4. Conclusions

The *A. quercifolia* rhizomes and leaves GC-MS profiles were established and revealed the plant nutritional value. Almost all the compounds herein reported were found for the first time in the species. The leaves richness in PUFA should be highlighted not only because it attests to their nutritional value but also will incentive its use in Timorese cuisine. The rhizomes richness in terpenoids should also be emphasized, particularly the cycloartane derivatives, compounds involved in phytosterols’ biosynthesis, which validate this plant’s use of rhizome to incentive the production of milk by young moms. The hopanoid derivatives herein revealed for the first time in this species, although common in ferns, may differentiate the species.

## Figures and Tables

**Table 1:** Dry weight of *A. quercifolia* and added volume of the solvent.

Part of Plant	DW (g)	V (mL)	HeW (g)	PyHe (%)
Leaves	10.01 ± 0.05	300.0 ± 0.1	0.58 ± 0.03	5.79 ± 0.09
Rhizomes	10.03 ± 0.05	300.0 ± 0.1	0.37 ± 0.01	3.69 ± 0.05

DW = Dry weight of the plant part used; V = Volume of *n*-hexane added; HeW = Hexane extract weight; PyHe = Percentage yield of the hexane extract.

**Table 2:** Linearity (y = mx + b, where y corresponds to the standard peak area/internal standard peak area ratio and x corresponds to the mass of standard/mass of internal standard ratio), LOD and LOQ of pure compounds used as reference.

Standard Compound	Slope (m) ^§^	Intercept (b) ^§^	R^2^	LOD ^§§^	LOQ ^§§^
Palmitic acid	0.2143	0	0.9944	15	50
1-Monopalmitin	7.2283	−0.0009	0.9975	3	10
Glycerol	7.2366	−0.0037	0.9937	3	10
Triacontane	2.0154	−0.0311	0.9991	10	33
Maltose	4.1401	−0.0801	0.9998	3	10
Mannose	4.1380	−0.1126	0.9999	5	17
β-Sitosterol	2.5254	−0.0033	0.9983	12	40
α-Tocopherol	2.4738	−0.0028	0.9993	5	17

^§^ in area counts mg^−1^; ^§§^ in µg/mL.

**Table 3:** Identified compounds on the *n*-hexane extract of *A. quercifolia* rhizome and leaves.

Identification *	Rt (min)	RI_NIST_	RI_cal_	Rhizome **	Leaves **
Carboxylic acids and derivatives					
Butanedioic acid ^b,c,d^	8.26	1170	1171	-	tr
Undecanoic acid ^b,c,d^	9.55	1704	1704	-	21.78 ± 0.19
Malic acid ^a,b,c^	9.86	1390	1392	-	tr
L-Glutamic acid ^a,b,c^	10.30	-	-	-	9.87 ± 0.23
Shikimic acid ^a,b,c^	13.14	1904	1904	-	28.58 ± 0.08
Citric acid ^a,b,c^	13.26	1944	1945	-	tr
Quininic acid ^a,b,c^	13.66	-	-	-	tr
Myristic acid	13.83	1788	1787	-	tr
3,4-Dihydroxyhydrocinnamic acid ^a,b,c^	14.96	1964	1962	-	tr
Dodecanedioic acid ^b,c,d^	15.42	1965	1966	-	tr
Glucaric acid ^a,b,c^	15.58	2249	2250	-	tr
Oct-3-enoic acid ^b,c,d^	16.26	1200	1202	-	15.67 ± 0.09
Palmitic acid ^a,b,c^	16.40	1987	1987	107.65 ± 0.12	237.27 ± 0.59
Linoleic acid ^a,b,c^	18.63	2202	2202	60.97 ± 0.08	153.81 ± 0.11
Linolenic acid ^a,b,c^	18.73	2210	2211	-	253.71 ± 0.93
Oleic acid ^a,b,c^	18.80	2194	2192	12.33±0.05	14.48 ± 0.24
Stearic acid ^a,b,c^	19.05	2186	2184	17.00 ± 0.02	15.49 ± 0.08
Arachidonic acid ^a,b,c^	20.72	2417	2415	-	19.13 ± 0.04
Oleoamide (9-Octadecenamide) ^a,b,c^	20.84	2228	2230	-	13.12 ± 0.03
Monopalmitin ^a,b,c^	23.63	2581	2583	0.50 ± 0.01	-
Lignoceric acid ^a,b,c^	27.75	2782	2783	-	tr
Terpenoids					
Neophytadiene ^b,c,d^	13.76	-	1832	-	tr
Squalene ^b,c,d^	27.39	2914	2910	2.59 ± 0.01	-
Cycloeucalenol acetate derivative ^b,c,d^	32.13	-	2909	14.61 ± 0.02	-
Stigmastan-3,5-diene ^c,d,e^	32.47	2525	2526	tr	-
α-Tocopherol ^a,b,c^	32.99	3226	3227	6.89 ± 0.01	-
Cycloeucalenol acetate ^b,c,d,§^	33.29	2900	2901	tr	tr
Serratene ^b,c,d^	33.53	2744	2745	tr	-
Lupeol ^a,b,c^	33.76	2848	2845	tr	-
Hop-16-ene ^b,c,d^	34.34	3420	3421	166.45 ± 0.53	-
Cycloartenol acetate ^b,c,d^	34.58	2907	2906	9.49 ± 0.01	-
9,19-Cycloergost-24-en-3-ol acetate ^b,c,d^	34.70	2956	2957	14.58 ± 0.03	-
Cholest-5-en-3(α)-ol ^b,c,d^	35.07	2954	2955	16.49 ± 0.02	-
Lupenone ^b,c,d^	35.28	3483	3481	7.22 ± 0.01	-
Stigmasterol ^a,b,c^	35.48	2797	2796	9.44 ± 0.01	-
4,14-Dimethyl-9,19-cyclolanost-24(28)-en-3-ol ^b,c,d^	35.76	2760	2761	10.75 ± 0.01	-
γ-Sitosterol ^a,b,c^	36.05	2731	2731	12.11 ± 0.05	-
β-Sitosterol ^a,b,c^	36.60	2789	2789	50.76 ± 0.11	-
Hop-21-ene ^b,c,d^	36.67	2659	2659	6.23 ± 0.01	-
Diploptene [Hop-22(29)-ene] ^b,c,d^	37.06	-	2667	48.01 ± 0.13	-
Cycloeucalenone ^b,c,d^	37.81	-	2981	26.75 ± 0.02	-
9,19-Cyclolanost-23-ene-3,25-diol 3-acetate ^b,c,d,§^	38.62	3071	3070	12.99 ± 0.01	-
Hop-17(21)-ene ^b,c,d^	38.80	-	2672	14.99 ± 0.01	-
3-*O*-Acetyl-6-methoxycycloartenol ^b,c,d^	39.01	3093	3091	5.94 ± 0.02	-
Cyclolaudenol ^b,c,d^	39.21	2834	2834	19.32 ± 0.06	-
Campesterol ^a,b,c^	39.62	2689	2685	25.64 ± 0.12	tr
31-Norcyclolaudenone ^b,c,d,§§^	40.43	-	3095	21.05 ± 0.06	-
Alcohols					
Glycerol ^a,b,c^	7.74	-	-	-	0.39 ± 0.01
Pentitol ^b,c,d^	12.13	-	-	-	0.95 ± 0.01
Phytol ^b,c,d^	18.07	2086	2086	-	16.43 ± 0.05
Alkanes					
*n*-Docosane ^e^	25.56	-	-	-	5.02 ± 0.02
*n*-Octacosane ^e^	28.91	-	-	-	38.05 ± 0.03
*n*-Tritetracontane ^b,c,d^	32.48	-	-	-	131.35 ± 0.64
*n*-Hentriacontane ^b,c,d^	36.06	-	-	-	13.04 ± 0.05
Carbohydrates					
D-Psicofuranose ^b,c,d^	12.99	2029	2029	-	1.73 ± 0.02
D-Tagatose ^b,c,d^	14.03	1982	1980	-	tr
D-Galactose ^a,b,c^	14.11	1970	1973	-	tr
D-Glucose ^a,b,c^	15.23	2037	2035	-	5.76 ± 0.04
Sucrose ^a,b,c^	23.86	3552	3551	1.97 ± 0.01	-

RT = retention time; RI_NIST_ = NIST14 mass spectral data retention index; RI_cal_ = retention index relative to *n*-alkanes (C_5_–C_36_); MV = mean value; SD = standard deviation; - = not found; tr = traces; * all compounds possessing hydroxy groups are identified as the correspondent TMS derivatives. Compounds were identified by: ^a^ comparison with pure silylated standards, ^b^ comparison with the GC-MS spectral libraries NIST14.lib and WILEY229.lib, ^c^ comparison with spectra found in the literature, ^d^ interpretation of MS spectrum fragmentation pattern; ^e^ comparison with pure standards; ** Values in MV ± SD; ^§^ (3β,4α,5α,9β)-4,14-dimethyl-9,19-cycloergost-24(28)-en-3-yl acetate; ^§§^ 4-Monomethylcycloartane.

## Data Availability

Not applicable.
